# How do caregivers of children with congenital heart diseases access and navigate the healthcare system in Ethiopia?

**DOI:** 10.1186/s12913-021-06083-2

**Published:** 2021-02-01

**Authors:** Sugy Choi, Heesu Shin, Jongho Heo, Etsegenet Gedlu, Berhanu Nega, Tamirat Moges, Abebe Bezabih, Jayoung Park, Woong-Han Kim

**Affiliations:** 1 Department of Health Law, Policy & Management, Boston University School of Public Health, Boston, MA USA; 2 Program in Global Surgery and Implementation Science, JW LEE Center for Global Medicine, Seoul National University College of Medicine, Seoul, Republic of Korea; 3 Seoul National University College of Nursing, Seoul, Republic of Korea; 4 National Assembly Futures Institute, Seoul, Republic of Korea; 5 School of Medicine, Addis Ababa University, Addis Ababa, Ethiopia; 6 Department of Thoracic and Cardiovascular Surgery, Seoul National University College of Medicine, Seoul, Republic of Korea

**Keywords:** Pediatric cardiac surgery, Caregiver decision-making, Patient experiences, Congenital heart disease, Child health, Health-care seeking, Ethiopia, Service delivery

## Abstract

**Background:**

Surgery can correct congenital heart defects, but disease management in low- and middle-income countries can be challenging and complex due to a lack of referral system, financial resources, human resources, and infrastructure for surgical and post-operative care. This study investigates the experiences of caregivers of children with CHD accessing the health care system and pediatric cardiac surgery.

**Methods:**

A qualitative study was conducted at a teaching hospital in Ethiopia. We conducted semi-structured interviews with 13 caregivers of 10 patients with CHD who underwent cardiac surgery. We additionally conducted chart reviews for triangulation and verification. Interviews were conducted in Amharic and then translated into English. Data were analyzed according to the principles of interpretive thematic analysis, informed by the candidacy framework.

**Results:**

The following four observations emerged from the interviews: (a) most patients were diagnosed with CHD at birth if they were born at a health care facility, but for those born at home, CHD was discovered much later (b) many patients experienced misdiagnoses before seeking care at a large hospital, (c) after diagnosis, patients were waiting for the surgery for more than a year, (d) caregivers felt anxious and optimistic once they were able to schedule the surgical date. During the care-seeking journey, caregivers encountered financial constraints, struggled in a fragmented delivery system, and experienced poor service quality.

**Conclusions:**

Delayed access to care was largely due to the lack of early CHD recognition and financial hardships, related to the inefficient and disorganized health care system. Fee waivers were available to assist low-income children in gaining access to health services or medications, but application information was not readily available. Indirect costs like long-distance travel contributed to this challenge. Overall, improvements must be made for district-level screening and the health care workforce.

**Supplementary Information:**

The online version contains supplementary material available at 10.1186/s12913-021-06083-2.

## 1. Background

As the World Health Organization (WHO) recognized the global burden and threat of non-communicable diseases (NCD) as one of the major challenges of the twenty-first century, increasing access to surgical care is becoming an increasingly important global health agenda due to its potential to save lives, prevent disabilities and decrease the burden of diseases [1, 2]. Congenital heart disease (CHD) is the leading cause of morbidity in children, [3] and about 500,000 children are born with CHD each year in Africa [4]. In a recent study, heart failure accounted for about 11% of pediatric admissions at a referral teaching hospital in Ethiopia [5]. Poverty, high fertility rates, and limited access to care add to the burden of CHD in LMICs [4]. As a result, research on cardiac surgical care capacity and demand is being actively conducted in the area [6].

Heart diseases require advanced diagnostic skills and delicate surgical procedures, which require a well-organized health system. Prior research shows that the Ethiopian health care system does not have enough medical personnel to diagnose CHD [7]. In addition to an inefficient health care system, the cost of preventing and treating heart diseases in Ethiopia poses a great burden on households [8]. Further, CHD negatively impacts family members financially and in their daily lives, leading to a significant decrease in quality of life [9]. Specifically, the burden of having open-heart surgery on a young child brings significant emotional strains for the caregivers. Without the right surgical care, a child’s neglected and exacerbated conditions may lead the caregiver to feel hopeless and distressed [10].

Timing of diagnosis is a problem in developing countries, where surgery is often delayed compared to the care in high-income countries [11]. CHD is not a disease that can be operated at any time. Many CHD can no longer be cured if the diagnosis and surgery is missed or delayed. In some cases like hypoplastic left heart syndrome or transposition of great arteries require surgery during the neonatal period within 1 month of birth [12]. In the case of CHD, delay in surgery raises the risk of bleeding during the surgery and requires higher precautions for infection prevention during post-operative care. It also increases the incidence of complications such as endocarditis and heart failure [13]. Because of this, patients who have CHD in Low- and middle-income countries (LMICs) may require more medical support and care compared to patients in other countries. This is because caring for patients with CHD can be complex and may require continuity of care, such as regular outpatient visits and drugs [14]. Also, barriers in low-income countries for caregivers are likely to be higher [15–17].

While the literature has clearly shown the limited access to pediatric cardiac surgery [5, 8, 18], little is known on the details of caregivers’ experience. Since caregivers are involved in surgical decision making for their child, it is important to assess and understand the healthcare system navigation process from the caregiver’s perspective. We conducted a qualitative study to understand the experiences of caregivers of children with CHD accessing the health care system and pediatric cardiac surgery. This was an exploratory analysis of data collected through interviews of caregivers of children who received surgical care at the public tertiary hospital in Addis Ababa. This study aims to offer ways to alter the design of health services toward greater patient-centeredness, thereby improving access to pediatric cardiac surgical care.

### 1.1. Conceptual framework

Conceptualizing an individual’s or a population’s health care utilization behavior is complex and multifaceted. One of the facilitators of such behavior is how patients decide to seek care based on the perceived needs for health services. Many frameworks have been conveyed as ways of conceptualizing how people come to think of themselves as ill or in need of care. Candidacy is a dynamic and contingent model that describes “the ways in which people’s eligibility for medical attention and intervention is jointly negotiated between individuals and health services” [19]. To explore Ethiopian caregivers’ access to health care utilization, we used Dixon-Woods et al.’s candidacy model which proposes the following elements: candidacy, navigation, the permeability of services, appearances at health services, adjudications, offers and resistance, operating conditions, and the local production of candidacy [19]. We used the candidacy framework because it allowed the understanding of the non-linear pathways to treatment. Identification of candidacy can be defined as how people recognize their symptoms as needing medical attention or intervention. It is the first step in accomplishing access to health care services. Navigation is the second step where individuals have to mobilize their resources to access the health care system. The permeability of services refers to how easily individuals can navigate through the services. Appearances at health services denote how individuals present themselves at the point of service. Adjudication refers to professional judgments and decisions that allow or inhibit the progression of candidacy. It pertains to patients’ perceived needs and their relationship with evaluated needs. Meanwhile, offers and resistance demonstrate how individuals may refuse offers from professionals. Finally, operating conditions and the local environment can also influence the relationships between practitioners and patients [19].

## 2. Methods

A qualitative study was conducted as part of a pediatric cardiac surgery capacity-building evaluation study at a teaching hospital in Addis Ababa, Ethiopia. We used the consolidated criteria for reporting qualitative research (COREQ) when preparing the manuscript. (Supplementary Material 1). At the time of the study, an annual in-country training was held to perform pediatric cardiac surgeries by the host country team and the team from the JW LEE Center for Global Medicine at Seoul National University College of Medicine. This study was conducted as a part of the project evaluation to identify areas for improvement. The study received ethical approval from the Addis Ababa University, College of Health Sciences Institutional Review Board (052/18/pedi) and the Seoul National University Hospital Institutional Review Board (1806–008-948).

In-depth face-to-face interviews were conducted by the first and second authors in December 2019 in Amharic then translated into English and Korean using trained local interpreters. We used convenience sampling to conduct interviews with 13 caregivers of all 10 patients with CHD that received cardiac surgery during the week of the interview. Interviews were offered only after the surgery had been completed and when children were placed into the intensive care unit. No caregivers selected for the interview refused to participate. The consent process stressed the nature of voluntary participation and how some parts of the interview were independent of the project evaluation itself. All participants provided oral and written consent for the involvement in the study. A small gift ($5 value) was given to the participants after the interview to compensate for their time.

The interviewers used the semi-structured interview guide set out to answer the caregiver’s health care navigation process from when they first noticed the symptoms of the child until the receipt of the current surgery (Supplementary Material 2). The guide was developed by our team of researchers using the candidacy framework. We were especially interested in exploring which service location they visited, the diagnosis process, referrals to treatment processes, and other barriers to care they encountered. All of the interviews were audio-recorded and lasted between 30 to 60 min. After the interview, we conducted medical chart reviews to compare data. This type of triangulation enabled us to validate the interview as well as add texture and background to the descriptions of care-seeking for this population. We experienced data saturation by the sixth interview, as no new data were emerging [20]. Data were transcribed verbatim by the second author and a research assistant and assessed by the first and third authors for completeness. The authors manually analyzed the transcripts according to the principles of interpretive thematic analysis, informed by the candidacy framework. A list of codes was developed by two coders (first author, second author) independently. Team meetings were held to discuss any differences in coding. The initial results from the analysis were presented at AcademyHealth 2020 and published as a selection of abstracts accepted for oral presentation [21].

## 3. Results

### 3.1. Study population

A total of 13 caregivers of 10 patients were interviewed (Table 1). Children were aged between one and 14 years old. Some interviews were attended by a single parent, mostly mothers, or both parents. Some patients were diagnosed with Tetralogy of Fallot, atrial septal defects (ASD), and ventricular septal defect (VSD). Many had multiple diagnoses. A complete list of diagnoses and procedures performed can be found in Supplementary Material 1.

**Table 1:** Characteristics of patients and the interview (*n* = 10)

Characteristics	n or range
Female	*n* = 5
Age range	1–14 yr old
Pre-operative weight	5–57 kg
Mother was present for the interview	*n* = 7
Both parents were present for the interview	*n* = 3

### 3.2. Identification of candidacy

Some caregivers mentioned that they learned about their child’s CHD right after newborn screening tests. However, if the child was not diagnosed soon after birth, caregivers could not recall if any screening was done. Also, not all children were born in a health facility. We examined that many misdiagnosed cases were correctly diagnosed after three to five, sometimes ten years. For those who did not find out right after birth, caregivers described how they came to recognize the symptoms of CHD including but not limited to fever, cough, shortness of breath, and vomiting. However, children with mild symptoms were thought to have a cold, and some parents did not perceive it as requiring formal medical assistance. Since some symptoms of CHD resemble a cold or flu, in many cases, children did not receive care for more than 5 years. Recurring symptoms prompted caregivers to seek formal health care. All of those who had home births noticed the symptoms but did not recognize them as CHD.

*“[He] was sick 4 years ago, but at that time, I didn’t know if his heart was sick. He was taking another medicine at the time.” (Father 3).*

Caregivers recalled during the interview that their children did seem a bit “strange” when they were babies and related past intense crying and screaming as additional signs of CHD.

*“[Before the diagnosis], she screamed and cried a lot, and had a lot of fever ... since birth.” (Father 5)*

*“[She] sweated a lot and she had shortness of breath as if she had a cold. She was sick since she was born, but when she got a cold, I took her to the hospital and she was prescribed with another medicine for lung problems. After a little more time and her conditions got worse, I realized it was a heart disease when she was 5 years old.” (Mother 9).*

### 3.3. Navigation experiences

Navigation experiences showed financial hardships, and the extra non-healthcare costs made treatment access even more challenging. However, we also observed strong social capital – many of the caregivers were able to borrow money from their family members and neighbors. Some caregivers followed their families’ or neighbors’ suggestions when choosing hospitals.

*“The most difficult thing about all this is money.”* (Mother 6).

*“Sometimes, when I don’t have money, I only buy enough [medications] for one month.”* (Mother 4).

*“We borrowed money from our family and neighbors. Thank God …*” (Mother 8).

Caregivers were not initially aware of CHD-specific services. Some caregivers were referred to multiple hospitals before reaching the hospital that offered surgery. Because most of the children were misdiagnosed with a cold or unknown disease at the time, caregivers had a difficult time choosing where to get care. The nearest public health center was the first point of care, but as symptoms persisted and exacerbated, caregivers started seeking alternative services such as private clinics and hospitals. Many caregivers went to multiple health centers and hospitals before getting referred to the current hospital.

Caregivers faced financial and practical barriers. First, caregivers faced costs for transportation, check-ups, consultations, and medications, and also needed to take time off work for appointments.

*“Because of breastfeeding, we have to travel together in a taxi and close our store to come to the hospital … It costs a lot.”* (Father & Mother 2).

At the current hospital, many had their consultation fees waived by providing a letter from their residency that showed financial hardship. These letters have to be renewed yearly. Some caregivers mentioned that they were not aware of such benefits and they were not able to go back to their town office to get that letter since they live far away from Addis Ababa.

### 3.4. The permeability of services

The permeability of services refers to how easily patients can access services. All of the caregivers had navigated the complex referral system to the teaching hospital. Caregivers felt that health centers and hospitals were relatively permeable for follow-up appointments once a CHD diagnosis had been given. However, accessing surgical care was not easy. Some caregivers have waited for more than 3 years. After two to three visits, caregivers were told to indefinitely wait for the surgery. One caregiver was given an option to send her child abroad for the operation.

“*Doctors ordered the operation to be performed in a foreign country because my child’s condition was serious. At first, I was sad that my child would never return if she goes abroad. It was especially difficult when they asked for my signature to take the baby. It was difficult [decision] at that time but I decided to wait longer …”* (Mother, 7).

Many caregivers described the helplessness they felt, mostly due to unpredictable surgery accessibility and indefinite waiting times.

### 3.5. Appearances at health services

Caregivers received services for their children through the diagnosis, medications, follow-up visits, and emergency care. Children who were born at home were more likely to experience a delay in diagnosis. Interviews indicated that time from birth to diagnosis was short if not misdiagnosed, but the time from diagnosis to treatment was long. Many caregivers mentioned how their child’s cold misdiagnosis contributed to the delay in CHD diagnosis. Caregivers also perceived it as a “mere cold” at the time. Many visited public health centers and public and private hospitals with recurring symptoms. Many described their frustration navigating multiple services, but some mentioned that being diagnosed and finally being able to receive a tangible treatment-like operation helped them.

“*There are a lot of people here, so they asked me to go to a private hospital nearby for a lab test,*

*and I paid a lot of money there.”* (Father, 9).

“*It was hard when I was going to several hospitals, but I felt better when I came here and learned that she can get surgery.”* (Father, 3).

### 3.6. Adjudications, offers, and resistance

Due to the uncertainty and lack of clear guidelines for CHD care, many caregivers were frustrated with healthcare experiences. They seemed to be vulnerable to adjudications during the process of seeking a CHD diagnosis, and upon receiving the diagnosis, had to wait for the operation with ambiguous advice about what to do next. Some parents were told there was nothing to do except wait for an operation. This quote also shows the caregiver’s resistance to the surgeon’s offer to seek medical care abroad. The last part of the quote demonstrates the recursive nature of the candidacy model as well.

“*It was especially difficult when they asked for my signature to take the baby. It was difficult [decision] at that time but I decided to wait longer …”* (Mother, 7).

*“At first, I went to a health center, and after a week, her conditions got worse, so I went to a.hospital and got medicine. This time, a big hospital, and private.”* (Father, 5).

Little resistance was identified during the interview, and many caregivers upheld providers’ recommendations and referrals. Due to expensive medications, some caregivers could not afford medications as recommended.

*“I only bought a 1 month supply instead of 3 because I could not afford it.”* (Mother, 4).

### 3.7. Operating conditions and the local production of candidacy

CHD care was not available outside Addis Ababa, and a referral is required to visit the teaching hospital. For this reason, until caregivers met the physician who referred them, treatment plans for CHD, especially surgeries, would not be feasible. As there is only one public teaching hospital where children can be given surgery for CHD, CHD operation was geographically inaccessible to caregivers who lived outside Addis Ababa. Many caregivers traveled across the country on a bus that takes several hours to get to Addis Ababa. Without an adequate place to stay in Addis Ababa, some participants slept in the hallways of the hospital.

## 4. Discussion

This study explored how caregivers in Ethiopia navigated the healthcare system for their children who have CHD, using the candidacy framework. It also elicited the challenges faced by caregivers when seeking treatment in a complex health system. Some caregivers reported that they were advised by their family members and neighbors about decision-making around navigating the healthcare system for their child. Caregivers reported that they frequently turned to advice from their family members or neighbors to pursue treatment for their child. It may be due to the lack of reliable information or lack of awareness and knowledge about CHD, available services, and locations of the services.

Major care-seeking delays were related to the inefficient and complex health care system, largely due to delayed CHD diagnosis, financial hardships, and a lack of hospitals that can perform operations. These findings are important given that caregivers of children who underwent cardiac surgery are under great psychological pressure. CHD surgery has a major impact on the financial status of the family, as well as their emotions and well-being [22]. However, it is important to note that caregivers described that being scheduled for surgery induced anxiety but also hope. This study provides an opportunity to obtain information from the perspective of this understudied population on how to improve the health care system for patients and their families.

Our results are in line with previous literature stating that birth typically occurred at home or in centers that did not have diagnostic capabilities, and that many caregivers delayed seeking care until children developed critical symptoms [11, 15]. Caregivers in our study reported that CHD of their child was misdiagnosed multiple times prior to seeking care at a large hospital and patients were waiting for the surgery for more than a year. CHD care in LMICs is not easily accessible for caregivers [15, 17, 22]. Many visited multiple services and were not able to access surgical services. Participants had to travel to Addis Ababa to receive the correct diagnosis as well as the surgery; however, most of the caregivers who had to travel long distances feared that potential medical complications may occur while transporting to the faraway hospital. Literature also reports a similar finding that caregivers living in rural areas felt discouraged visiting a faraway hospital in urban areas [23]. This may be why caregivers delay the first consultation with a doctor at a tertiary hospital [11].

This study provides an opportunity to obtain information from the caregivers’ perspective to systematically improve access to CHD care. As Dixon-woods et al. hypothesize, vulnerable populations such as patients with CHD live in deprived circumstances, and caregivers are therefore likely to manage health issues as a series of catastrophes. Perceived benefits are likely to be low if the permeability of services is low. Easing the navigation process also requires health literacy regarding the available services. By trying to increase the permeability of treatment services, the system can improve patient-centeredness by enhancing patients’ experiences and ultimately changing their perceptions of CHD treatment.

At the system level, the pediatric workforce shortage is a significant barrier to increasing access to surgical care [23–25]. Along with increasing health care workforce capacity for pediatric cardiac surgeries in Ethiopia, there is a need to strengthen the district-level screening capacity to facilitate earlier diagnosis at easily accessible health care settings. Our study reveals that there is a lack of clear guidelines for pediatric CHD surgical care at primary care and secondary-level hospitals. A referral is required to visit the teaching hospital, which can lead to a delay in care. Such a barrier can lead to poor outcomes as complications and co-morbidities [15]. Additionally, currently in Ethiopia, newborn screening for CHD is not done routinely with a pulse oximeter or echocardiography. Finally, educating pediatric cardiologists and cardiac surgical teams on diagnostic and surgical care is also important to allow the development of pediatric cardiac care in Ethiopia. Ethiopia needs to prioritize policies that protect the financial status of low-income households that need health care services. Financial protection is low despite the availability of fee-waivers for medications. The government should better publicize and provide fee-waivers for patients in need.

There are some important limitations to our study. The sample size was small and due to purposive sampling including caregivers whose children had surgery, our results may not be generalizable to all caregivers of children who have CHD in Ethiopia. Although the focus of this study was to investigate the experience of those who managed to successfully navigate the health care system, we were not able to address the perspectives of individuals who were unable to navigate it successfully. Future studies must take this into consideration and examine barriers experienced by unsuccessful navigators who we were not able to identify in our study. There are likely much worse barriers related to accessibility, perceptions, knowledge, beliefs, or cultures that may affect access to pediatric cardiac surgical care among caregivers who are unable to navigate the health system [26]. Another limitation is that there may be variations in findings depending on the availability of existing community resources where caregivers reside. Since caregivers were asked to remember their prior care-seeking behaviors, the results may have been influenced by recall bias. To minimize this, we conducted chart reviews, and the interviewers probed for further explanations. During the interview, both English and Korean were used by the interpreter. However, qualitative nature was particularly useful for highlighting the pathways and experiences of caregivers. Finally, this study presents the views of the patients through their caregivers, thus, future studies should explore providers’ experiences with diagnosing, referring, treating, and operating for children who have CHD in low-resourced settings.

## 5. Conclusions

Overall, caregivers had financial difficulties and struggled in the disorganized Ethiopian health care system, experiencing poor service quality and inaccurate diagnoses. These interviews provide a greater understanding of the complex Ethiopian health care system, and the experience of patient caregivers will help to further develop future research and programs to improve pediatric cardiac surgical care in LMICs.

## Supplementary Information

**Additional file 1.** Consolidated criteria for reporting qualitative studies (COREQ).

**Additional file 2. **Semi-structured interview guide.

## Data Availability

The datasets used and/or analyzed during the current study are available from the corresponding author on reasonable request.

## Abbreviations

CHD: Congenital heart disease
CVD: Cardiovascular disease
LMIC: Low- and middle-income countries
NCD: Non-communicable diseases
WHO: World Health Organization
