# Supplementary material for: How do caregivers of children with congenital heart diseases access and navigate the healthcare system in Ethiopia?
Source: BMC Health Serv Res. 2021 Feb 1;21:110. doi: 10.1186/s12913-021-06083-2 (PMC7852139; doi:10.1186/s12913-021-06083-2)
Supplement: Supplementary file 1 — Additional file 1. Consolidated criteria for reporting qualitative studies (COREQ). [file 12913_2021_6083_MOESM1_ESM.docx]

**Supplementary Material 1. Consolidated criteria for reporting qualitative studies (COREQ)**

**Domain 1: Research team and reflexivity**

**Personal Characteristic**

1. Interviewer(s)/facilitator(s): The first author and the second author.
2. Credentials: The first author MS, PhD candidate and the second author RN
3. Occupation: The first author was in PhD study and the second author was research associate
4. Male or female: The first author is female and the second author is male
5. Experience and training: Both have training in interviewing
6. Relationship established: Yes, both researchers built relationships prior to the study
7. Participants’ knowledge of the interviewer: Before the interviews, participants received information about the study and the reason for doing the study. Health professionals (doctor or nurse) explained the study.
8. Interviewer characteristics: Both interviewers were interested in assessing access to health care services in developing countries. We discussed our preunderstanding during the interview process with the other authors in the group.

**Domain 2: Study design**

**Theoretical framework**

1. Methodological orientation and Theory: Candidacy framework by Dixon-woods (pg 5-6)

**Participation selection**

1. How were participants selected? Convenience sampling, we selected caregivers of patients who received cardiac surgery during the week of the visit
2. Method of approach: face to face by their doctor or nurses
3. Sample size: 13 caregivers
4. Non-participants: None

**Setting**

1. Setting of data collection: All interviews were conducted in a suitable room in the hospital.
2. Presence of non-participants: None
3. Description of sample: See table 1 as well as the table below.

| No. | Diagnosis | Sex | Age | Weight (kg) | Surgical Procedures Performed | Interviewee |
| --- | --- | --- | --- | --- | --- | --- |
| 1 | PS  ASD  PDA | FEMALE | 4y 9m | 15.6 | PS Relieve ASD Primary Closure PDA Division | Mother, Father |
| 2 | VSD ASD  DCRV  PULMONARY HYPERTENSION | MALE | 3y 3m | 9.5 | VSD Patch closure ASD Primary Closure RV Infundibular Muscle Wide Resection | Mother, Father |
| 3 | TOF | MALE | 14y | 57 | TOF Total correction | Father |
| 4 | VSD MR Pulmonary Hypertension VSD Subaortic Diffuse Fibrotic Ridge MR RV hypertrophy Pulmonary hypertension | FEMALE | 3y 1m | 8.8 | VSD Patch Closure MR Repair RV EFE Wide resection Hypertrophic muscle resection PDA double ligation | Mother |
| 5 | TOF | MALE | 5y | 14 | TOF Total correction | Father |
| 6 | PDA AS Pulmonary Hypertension Severe heart failure | FEMALE | 1y 2m | 5 | PDA division | Mother |
| 7 | Pulmonary Stenosis ASD Squeezed LV RV dysfunction Pericardial Effusion | MALE | 1y 9m | 12 | Pulmonary Valve repair RA Reduction-plasty ASD Primary Closure RV Infundibular Muscle Wide Resection | Mother |
| 8 | Pulmonary Stenosis RVH ASD | MALE | 7y 2m | 25 | Pulmonary Valve replair ASD Primary closure RV Infundibular muscle wide resection | Mother |
| 9 | ASD  AR | FEMALE | 6y 7m | 17 | ASD Patch closure | Father |
| 10 | ASD MR | FEMALE | 4y | 15 | ASD Patch closure MR Repair | Father, Mother |

**Data collection**

1. Interview guide: See Supplementary Material 2
2. Repeat interviews: No.
3. Audio/visual recording: Interviews were audio-recorded.
4. Field notes: The second author made field notes during the interviews.
5. Duration: The focus groups interviews lasted between 30 and 60 minutes.
6. Data saturation: Yes, it was first discussed among the first and second author, and then with the research group. We experienced data saturation by the sixth interview.
7. Transcripts returned: N/A

**Domain 3: analysis and findings**

**Data analysis**

1. Number of data coders: The first and second author coded the data using the candidacy framework. All the researchers discussed the codes and subthemes.
2. Description of coding tree: Candidacy framework was used and we started journey-mapping the health care visits
3. Derivation of themes: Candidacy framework
4. Software: Excel
5. Participating checking: No

**Reporting**

1. Quotations presented: Yes, we included participant numbers.
2. Data and findings consistent: Yes, there was consistency between the data presented and the findings.
3. Clarity of major themes: Yes
4. Clarity of minor themes: Yes
