# Supplementary material for: How do caregivers of children with congenital heart diseases access and navigate the healthcare system in Ethiopia?
Source: BMC Health Serv Res. 2021 Feb 1;21:110. doi: 10.1186/s12913-021-06083-2 (PMC7852139; doi:10.1186/s12913-021-06083-2)
Supplement: Supplementary file 2 — Additional file 2. Semi-structured interview guide. [file 12913_2021_6083_MOESM2_ESM.docx]

**Semi-structured interview guide**

**Caregiver interview for health system navigation – Ethiopia Dec, 2019**

**Surgery date:**

**Surgery completed at the time of interview: Yes / No**

**Gender: M / F**

**Relationship to the patient: Father / Mother / both / Grandparents / if others, specify:**

**Age:**

**Region:**

**Distance/time to the hospital?**

**1. Qualitative interview introduction**

Length: 30-45 minutes

Primary goal: To see things the way you see them… more like a conversation with a focus on your experience, your opinions and what you think or feel about the topics covered

**2. Background Information**

Overview:

Invite interviewee to briefly tell me about him/herself: General information about background… mostly about experiences and perspectives on issues surrounding pediatric congenital heart disease (CHD)

If the interviewee openly identifies diagnosis:

Diagnosis:

When:

**3. CHD experience** – (Identification, Navigation, Permeability of services, Appearances at health services, Adjudications, Offers and resistance, Operating conditions)

Can you tell me about your [baby’s name]’s experience with CHD?

(Ask about Identification)

• Where tested

- Where diagnosed

(Ask about Navigation)

• Was your experience positive or negative? Why?

• Linked to other services? Which ones?

• Change in behavior?

• Total number of prior visits to the doctor/hospital for CHD?

**4. Pediatric Cardiac Surgery**

(Availability of cardiac surgery, Permeability of services, Appearances at health services)

• Have you been to other hospitals?

• Can you tell me about your experience? Positive or negative? Why?

• Where have you been?

• Barriers to care

• Economic burden? (Transportation, loss in income, costs of unpaid care, coping costs)

• Stigma? Health care workers attitude?

• Linked to other services? Which ones: Testing? Treatment? Other?

• Length of time:

• Ability to adhere to recommendations:

**5. Resource availability** (Operating conditions)

Is pediatric cardiac surgery available in your country?

• If yes, where?

• Quality of services?

• Accessibility, cost?

• Acceptability of service delivery?

• Knowledge of Treatment issues: has this affected your navigation?

• Closing: Any other comments/things we’ve missed?

**Next Step: Conduct chart review**
